# Extant primates and development of primatology in China: Publications, student training, and funding

**DOI:** 10.24272/j.issn.2095-8137.2018.033

**Published:** 2018-05-12

**Authors:** Peng-Fei Fan, Chi Ma

**Affiliations:** 1 School of Life Sciences, Sun Yat-Sen University, Guangzhou Guangdong 510275, China

**Keywords:** Gibbon, Snub-nosed monkey, Leaf monkey, Macaque, Slow lories

## Abstract

China supports the richest non-human primate diversity in the northern hemisphere, providing an excellent opportunity for Chinese primatologists to take a leading role in advancing the study of primatology. Primatology in China began to flourish after 1979. To date, Chinese primatologists have published more than 1 000 papers in journals indexed by the Chinese Science Citation Database and the Web of Science Core Collection, and universities and academic institutions have trained 107 PhD students and 370 Masters students between 1984 and 2016. In total, the National Science Foundation of China has funded 129 primate projects (RMB 71.7 million) supporting 59 researchers from 28 organizations. However, previous research has also shown obvious species bias. *Rhinopithecus roxellana*, *Rhinopithecus bieti*, and *Macaca mulatta* have received much greater research attention than other species. Researchers have also tended to continue to study the same species (55.2%) they studied during their PhD training. To promote the development of primatology in China, we suggest (1) the need for a comprehensive primatology textbook written in Chinese, (2) continued training of more PhD students, and (3) encouragement to study less well-known primate species.

## INTRODUCTION

China supports the richest diversity of non-human primates (hereafter primate) in the northern hemisphere. More than 20 species from three families currently reside in China (Table 1), with the discovery of new species and populations still adding to the list. Remarkably, two new species (*Hoolock tianxing*: Fan et al., 2017; *Macaca leucogenys*: Li et al., 2015) have been described and new populations of three species (*Nomascus nasutus*: Chan et al., 2008; *Trachypithecus pileatus*: Hu et al., 2017; *Rhinopithecus strykeri*: Long et al., 2012) have been discovered in China since 2006. In total, 32 primate species taxa have been reported in China by different researchers (Table 1). The Chinese population of eastern hoolock gibbon (*Hoolock leuconedys*), once thought to live in western Yunnan (Fan et al., 2011), is now recognized as a new species, known as the skywalker hoolock gibbon (*H. tianxing*: Fan et al., 2017). Consequently, there are currently no populations of eastern hoolock gibbon in China. Thus, excluding species with unconfirmed distribution (*Pygathrix nemaeus*, Pan et al., 2007) or disputed taxonomy (e.g., *Nycticebus* sp., Pan et al., 2007) and the two species occurring in Indian controlled areas of southeastern Tibet (*Trachypithecus geei* and *Hoolock hoolock*, Ji & Jiang, 2004; Jiang et al., 2017), there are 27 species of primates in China available for study (Table 1).

**Table 1:** Number of primate species of China in different literature

Species	1	2	3	4	This study	Protection level	English papers (*n*)	Chinese papers (*n*)	Masters thesis (*n*)	PhD dissertation (*n*)	No. of NSFC grants (*n*)	Total funding (RMB 10 000)
*Nycticebus* sp.		*√*										
*Nycticebus pygmaeus*	*√*	*√*	*√*	*√*	*√*	Class I	5	17	2	3	2	42
*Nycticebus bengalensis*	*√*	*√*	*√*	*√*	*√*	Class I	1	11	1	1	0	0
*Nomascus nasutus*			*√*	*√*	*√*	Class I	10	8	4	0	1	17
*Nomascus leucogenys*	*√*	*√*	*√*	*√*	*√*	Class I	0	1	4	0	1	23
*Nomascus hainanus*	*√*	*√*	*√*	*√*	*√*	Class I	14	11	3	4	2	113
*Nomascus concolor*	*√*	*√*	*√*	*√*	*√*	Class I	28	13	10	6	4	155
*Hylobates lar*	*√*	*√*	*√*	*√*	*√*	Class I	2	10	1	0	0	0
*Hoolock tianxing*				*√*	*√*	Class I	5	35	12	0	3	156
*Hoolock leuconedys*	*√*	*√*	*√*	*√*								
*Hoolock hoolock*				*√*								
*Trachypithecus shortridgei*	*√*	*√*	*√*	*√*	*√*	Class I	2	3	4	1	0	0
*Trachypithecus pileatus*				*√*	*√*	Class I	0	0	0	0	0	0
*Trachypithecus phayrei*	*√*	*√*	*√*	*√*	*√*	Class I	3	37	4	4	1	75
*Trachypithecus leucocephalus*	*√*	*√*	*√*	*√*	*√*	Class I	30	39	20	8	8	353
*Trachypithecus geei*	*√*											
*Trachypithecus francoisi*	*√*	*√*	*√*	*√*	*√*	Class I	12	111	42	4	8	258
*Trachypithecus crepusculus*			*√*	*√*	*√*	Class I	0	0	0	0	0	0
*Semnopithecus schistaceus*	*√*	*√*	*√*	*√*	*√*	Class I	0	9	1	1	0	0
*Rhinopithecus strykeri*			*√*	*√*	*√*	Class I	4	3	2	0	1	62
*Rhinopithecus roxellana*	*√*	*√*	*√*	*√*	*√*	Class I	135	215	112	37	33	2 051
*Rhinopithecus brelichi*	*√*	*√*	*√*	*√*	*√*	Class I	15	25	7	0	3	85
*Rhinopithecus bieti*	*√*	*√*	*√*	*√*	*√*	Class I	65	109	43	18	18	789
*Pygathrix nemaeus*		*√*										
*Macaca thibetana*	*√*	*√*	*√*	*√*	*√*	Class II	77	86	35	7	10	353
*Macaca munzala*			*√*	*√*	*√*	Not assessed	0	0	0	0	0	0
*Macaca mulatta*	*√*	*√*	*√*	*√*	*√*	Class II	37	353	73	24	16	1 239
*Macaca leucogenys*			*√*	*√*	*√*	Not assessed	3	1	0	0	0	0
*Macaca leonina*	*√*	*√*	*√*	*√*	*√*	Class I	12	14	6	0	0	0
*Macaca cyclopis*	*√*	*√*	*√*	*√*	*√*	Class I	35	8	29	5	0	0
*Macaca assamensis*	*√*	*√*	*√*	*√*	*√*	Class I	6	70	11	1	1	62
*Macaca arctoides*	*√*	*√*	*√*	*√*	*√*	Class II	4	59	4	0	0	0
Total	22	23	26	29	27							

1: Ji & Jiang, 2004; 2: Pan et al., 2007; 3: Jiang et al., 2015; 4: Jiang et al., 2017 and this study. This table also shows the species bias in research. Total funding for each species was incomplete because it was calculated from the database of the National Science Foundation of China. The State Forestry Administration of China, Ministry of Science and Technology of China, and other organizations also support primate studies, but we did not have access to this information.

Chinese primates live in diverse habitats and many populations represent the northern most distribution of the species, genus, or family. For example, *R. bieti* lives in dark coniferous forest above 3 000 m in Yunnan Province and Tibet; *Trachypithecus leucocephalus* survives in karst forests without surface water in Guangxi; *Nomascus concolor* ranges in mountain forests that can be covered by snow in winter in central Yunnan. In these harsh seasonal habitats, primates have evolved different strategies from their lowland conspecifics or close relatives to cope with ecological and social challenges, such as living in large groups. Snub-nosed monkeys (*Rhinopithecus* spp.) live in multi-level societies of up to 400 individuals (Qi et al., 2014) and leaf monkeys (*Trachypithecus crepusculus*) living in seasonal montane forests on Mt. Wuliang form multi-male multi-female groups of more than 100 individuals, whereas their low-land relatives normally live in one-male groups of less than 30 individuals (Fan et al., 2015a). Crested gibbons (*Nomascus nasutus*, *N. concolor*, and *Nomascus hainanus*) live in groups with two breeding females in seasonal forests in China, whereas other gibbon populations typically live in adult pair groups with only one breeding female (Fan et al., 2015b; Guan et al., 2018). Populations living in colder or more temperate habitats provide excellent opportunities to study evolutionary and ecological differences in population genetics, nutritional ecology, endocrinology, cognition, and microbiome.

At present, most primates in China are threatened by habitat loss and degradation, illegal hunting, and small population size (Fan et al., 2017; Liu et al., 2015). Except for *M. mulatta*, *Macaca thibetana*, *Macaca cyclopis*, and *R. roxellana*, all other species have total populations of less than 10 000 individuals (Li et al., in preparation). Two gibbon species have recently become extinct in China (*Nomascus leucogenys*: Fan et al., 2014; *Hylobates lar*: Grueter et al., 2009) and the Hainan gibbon has a total population of less than 30 individuals, being the most endangered primate species in the world (Bryant et al., 2016). According to the Chinese Wildlife Conservation Law, 22 species are listed as National Class I Protected Species, three species (*M. thibetana*, *M. mulatta*, and *Macaca arctoides*) are Class II, and two species (*Macaca munzala* and *M. leucogenys*) have not yet been assessed (Table 1). Conservation oriented research can provide an informed scientific foundation to support species protection and preservation.

Primatology is the scientific study of both living and extinct primates in either their natural habitats by field surveys or in laboratory experiments to understand aspects of their evolution and behavior (https://en.wikipedia.org/wiki/Primatology). Ji & Jiang (2004) dated the rise of primatology in China back to 1862 when Swinhoe published his paper about mammals from the island of Formasosa (Swinhoe, 1862); however, Chinese scientists first studied primates in the 1950s (Shou, 1957; Tan, 1957). Xia & Zhang (1995) and Ji & Jiang (2004) provided good summaries on the research and conservation of primates in China. Primatology, especially studies of wild populations in China, has experienced a strong development trajectory. Several teams have established field stations and conducted continuous long-term research for more than 20 years.

The Chinese Primatological Society was formally established in 2017. To celebrate this landmark event, we organized this special issue on “Primates and Primatology in China”, which includes studies on gibbons, leaf monkeys, snub-nosed monkeys, and macaques, as well as commentaries from Prof. Paul A. Garber and Prof. Colin A. Chapman. To provide a basic introduction to primates and primatology in China, we briefly review the development of primatology in China through three indices: that is, number of publications, student training, and funding received from the National Natural Science Foundation of China (NSFC). We also consider the current challenges facing primatology development in China. This review excludes studies on extinct species or on primates as medical models.

## MATERIALS AND METHODS

We searched for papers published in Chinese journals using the Chinese name of each extant species (including title, keyword, and abstract) within the Chinese Science Citation Database (CSCD) using Chaoxing, a Chinese literature database (http://www.chaoxing.com). We searched for papers published in international journals through the Web of Science Core Collection (WSCC) database (http://apps.webofknowledge.com) using the Latin name of each extant species and refined the results by confining the county/region to “China”, “Hong Kong”, or “Taiwan”. We searched for Masters theses and PhD dissertations in Chinese within several databases, including CNKI, Wanfang (http://www.wanfangdata.com.cn/), Airitilibrary (http://www.airitilibrary.cn), and Chaoxing (http://www.chaoxing.com/), using the Chinese name of each species as the search term. We searched for grants for each species funded by the NSFC since its establishment in 1986 (http://www.medsci.cn/sci/nsfc.do, http://nsfc.biomart.cn/). Although primate research has received funding from the State Forestry Administration of China, Ministry of Science and Technology of China, and other organizations, the NSFC is the largest funding source for research and the only open source to which we had access. We also used genus and family names when searching for papers published in Chinese and English, and for grants. We checked each paper and thesis and excluded those using primates as medical models. We also excluded grants for studies of more than one species (*n*=12) when determining total NSFC funding for each species. We calculated the proportion of international cooperation by dividing the number of papers in which an international organization was involved based on author affiliation by the total number of papers published. We also obtained anecdotal information from primatologists in the WeChat group “China Primatological Society” by asking “who teaches primatology-related courses to undergraduate students” and “who obtained a PhD degree aboard”. We analyzed data and constructed figures using Microsoft Excel 2010.

## RESULTS

### Number of publications

**Figure 1:**
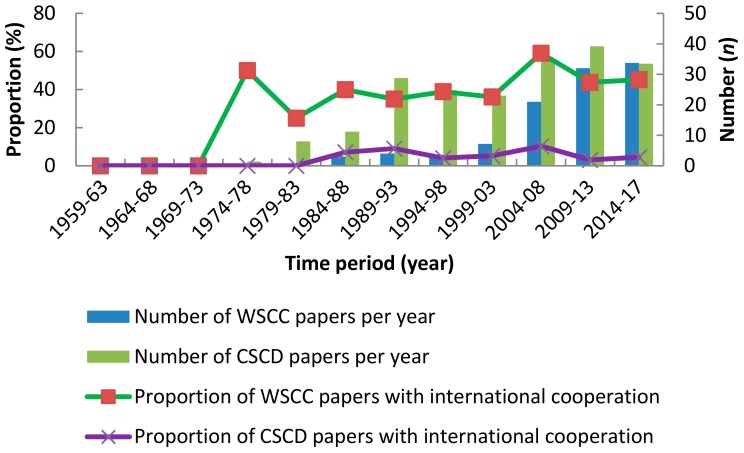
Number of papers published per year by Chinese authors

In total, we found 496 papers published by Chinese authors from the WSCC and 999 papers from the CSCD (http://www.chaoxing.com). Only 15 papers were published in CSCD journals and three papers in WSCC journals before 1979, after which primate studies began to flourish. We observed stable development of Chinese primatology between 1979 and 2003, with China then entering a new phase after the 2002 International Primatological Society meeting held in Beijing. After 2003, Chinese primatologists published a greater number of papers in international journals (WSCC database), and the number of papers in the WSCC database exceeded the number of papers published in the CSCD for the first time between 2014 and 2017 (Figure 1).

From the Web of Science Core Collection (WSCC: http://www.webofknowledge.com) and Chinese Science Citation Database (CSCD: http://www.chaoxing.com), and proportion of international cooperation during each time period (The 2017 database is not complete).

Chinese primatologists have also actively engaged in international cooperation since the rise of primatology in China. In total, scholars based at international universities and academic institutions have been involved in 228 English papers (46.0%, *n*=496). The proportion of international cooperation in WSCC papers has remained above 25% over time (Figure 1). In contrast, very few (3.3%, *n*=999) CSCD papers have included international scholars (Figure 1).

### Student training

**Figure 2:**
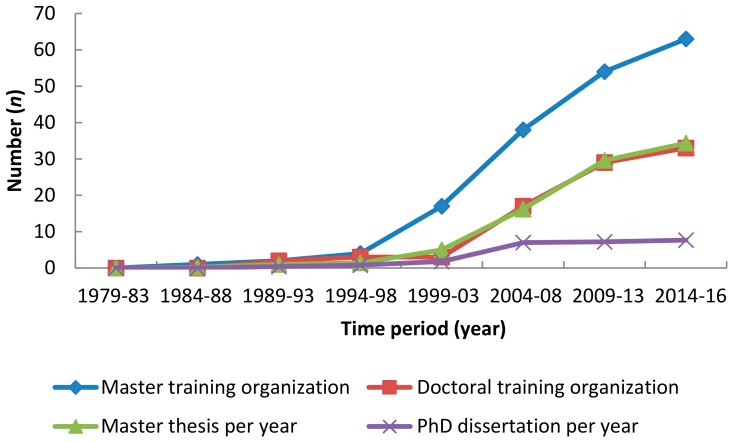
Average number of students per year that studied primates as part of their research, and the accumulated number of training organizations in China

China commenced student training in primate research after 1979. However, primatology is not a distinct discipline in China, with graduate students who have studied primates receiving their degrees in zoology, ecology, genetics, physiology, and anthropology. The first graduate student (Ya-Wen Huang) who studied the habitat of the Hainan gibbon obtained her Masters degree at Sun Yat-Sen University in 1984. The first PhD student (Ya-Ping Zhang) studied genetic diversity of the genus *Macaca* and obtained his PhD degree from the Kunming Institute of Zoology, Chinese Academy of Sciences (CAS) in 1991. Since then, there has been a dramatic increase in the number of graduate students studying primates (Figure 2). From 1979 to 2016, 109 graduate students have obtained a PhD and 370 students have obtained their Masters in primate-related research. From 2013 to 2016, an average of eight PhD students and 31 Masters students have received their degree each year (Figure 2). Based on information collected from the WeChat group “China Primatological Society”, six Chinese primatologists have obtained their PhD degree abroad.

The number of organizations that train Masters and PhD students in primate studies has also increased in China (Figure 2). Prior to 2000, only four organizations (Kunming Institute of Zoology (CAS), Peking University, Beijing Normal University, and Institute of Zoology (CAS)) trained PhD students and seven organizations trained Masters students in the study of primates. These numbers increased to 33 and 63, respectively, from 2014 to 2016 (Figure 2). Currently, however, only two professors teach primatology to undergraduate students in China.

### NSFC funding

**Figure 3:**
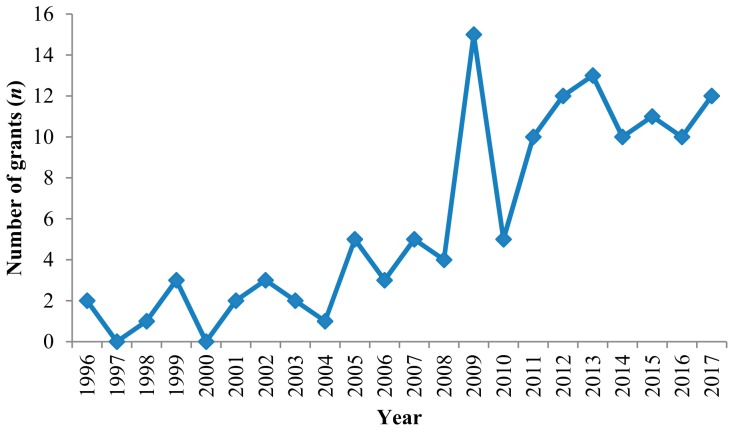
Number of grants related to primate research funded by the National Natural Science Foundation of China

**Figure 4:**
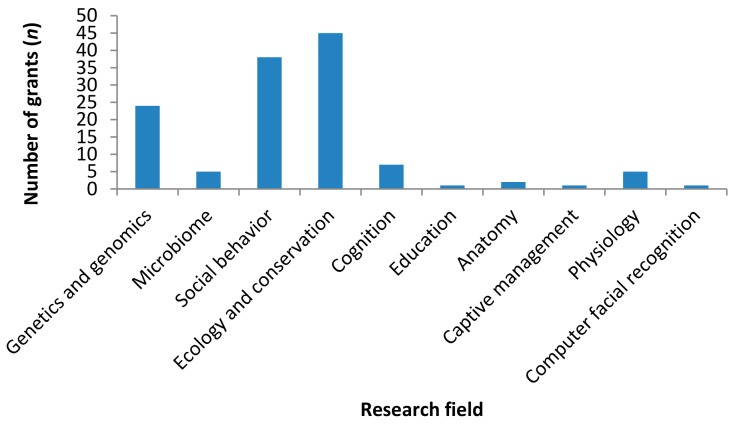
Research fields of the 129 NSFC-funded primate projects

Although the NSFC was established in 1986, it only funded its first primate field project in 1996 (study commenced in January 1997). Further studies were funded after 2009 (Figure 3). In total, the NSFC has funded 129 primate projects (RMB 71.7 million), supporting 59 researchers from 28 organizations. Of the 129 projects, 112 focused on a single species, 12 on a single genus, two at the family level, and three on the order Primates. Ecology and conservation, social behavior, and genetics and genomics are the three most common research fields, with several projects studying cognition, microbiome, anatomy, and physiology, and three focusing on education, computer facial recognition, and captive management (Figure 4).

## Species bias

We found an obvious species bias in primate research in China (Table 1). Two endemic snub-nosed monkey species (*R. roxellana* and *R. bieti*) and common *Macaca mulatta* have received more research attention in China than any other taxa. More students have studied these species as their thesis subject, more projects on these species have been funded, and more papers have been published. At the same time, other species such as *Trachypithecus crepusculus*, *Trachypithecus pileatus*, *Semnopithecus schistaceus*, and *Nycticebus bengalensis* have been completely overlooked (Table 1).

Thirty-three researchers who studied primates for their PhD received NSFC grants. However, except for a few senior professors, only two Masters degree holders have received grants from the NSFC. Researchers with a PhD have tended to continue studying the same species (55.9%), genus (70.6%), or family (85.3%).

## DISCUSSION

Chinese scientists have made significant progress in primate research and student training since 1979. However, both total funding and total number of PhD students remain small. China has only trained 109 PhD students since 1979, with less than eight PhD students defending their degree each year from 2014 to 2016. As Masters students have rarely been funded by the NSFC, and therefore have been unable to develop a successful research career, we must train and encourage more PhD students in order to promote primate research and conservation in China.

We also need to pay attention to the obvious species bias shown in current research. Some species like *R. roxellana* and *R. bieti* are well studied and their populations are stable or increasing, whereas species like *T. pileatus* and *N. bengalensis* have received little attention and are on the edge of extinction. A pattern of continuing to study the same species after PhD training has only exacerbated the situation. Therefore, researchers should be encouraged to study overlooked species or focus on inter-species comparisons, as most NSFC projects have focused on single species.

Another issue facing Chinese students is the lack of Chinese language textbooks on primatology. Currently, students must read English texts when learning and developing an interest in primatology, which can inhibit and discourage those with poorer English skills. Although many professors teach zoology or ecology in China, at present only Dr. Peng Zhang teaches primatology at Sun Yat-Sen University, and Drs. Dong-Po Xia and Bing-Hua Sun teach primatology at Anhui University. Consequently, systematic training in primatology for undergraduate and graduate students remains deficient in China, which can increase the difficulties for post-doctoral researchers to expand their area or species of interest. Given that developing a new discipline in China is challenging, we urgently need a Chinese language textbook that will encourage interested students to learn about primatology as undergraduates.

China has made significant progress in primatology since 1979. To promote further development, however, we need to establish a high-quality Chinese language primatology textbook, train more PhD students, and encourage post-doctoral researchers to study less well-known primate species in China. Furthermore, new students need to be inspired to study primates and public awareness of primatology and primate conservation should be increased. Chinese primatologists need to be encouraged to write popular science books and introduce primates during lectures and talks. We sincerely hope that primates, primatology, and primatologists will have a brighter future in China.
